# Supporting Family Caregivers of Nursing Home Residents with Dementia in Their Last Week of Life: A Survey Among Bereaved Family Caregivers

**DOI:** 10.1089/pmr.2024.0088

**Published:** 2025-03-05

**Authors:** Hinke E. Hoffstädt, Maartje S. Klapwijk, Iris D. Hartog, Yvette M. van der Linden, Bart J.A. Mertens, Arianne Stoppelenburg, Jenny T. van der Steen

**Affiliations:** ^1^ Center of Expertise in Palliative Care, Leiden University Medical Center, Leiden, the Netherlands.; ^2^ Department of Public Health and Primary Care, Leiden University Medical Center, Leiden, the Netherlands.; ^3^ University Network for the Care Sector Zuid-Holland, Leiden University Medical Center, Leiden, the Netherlands.; ^4^ Netherlands Comprehensive Cancer Organisation (IKNL), Utrecht, the Netherlands.; ^5^ Medical Statistics, Department of Biomedical Data Sciences, Leiden University Medical Center, Leiden, the Netherlands.; ^6^ Radboudumc Alzheimer Center and Department of Primary and Community Care, Radboud University Medical Center, Nijmegen, the Netherlands.; ^7^ Cicely Saunders Institute, King’s College London, London, UK.

**Keywords:** dementia, end-of-life care, family caregivers, nursing homes, palliative care

## Abstract

**Background:**

Family caregivers of people with dementia in nursing homes may need support from healthcare providers, especially when death is approaching.

**Objective:**

To increase our understanding of family caregivers’ experiences in their relative’s last week of life before and during the pandemic, their needs for emotional, practical, and spiritual support, and the extent to which those needs are accommodated by healthcare providers.

**Design:**

Survey among bereaved family caregivers of people with dementia recruited from six nursing homes in the Netherlands in 2018–2019 and 2020–2022.

**Results:**

The questionnaire was completed by 165 family caregivers (response rate: 55%). Most respondents (79%) rated the overall care they received as “excellent,” “very good,” or “good.” More respondents reported a need for emotional (74%) and practical (64%) support than for spiritual support (37%). Emotional and practical support were more commonly “always” or “most of the time” provided (63% and 51%, respectively) than spiritual support (22%). Differences existed in the presence of practical, emotional, and spiritual support needs (*p* < 0.001) and the frequency in which those support types were provided when there was a need (*p* < 0.001). The overall care that was received was more likely to be rated as “excellent” or “very good” when a higher frequency of emotional (*p* < 0.001), spiritual (*p* < 0.002), or practical (*p* < 0.001) support was reported. Before and during the pandemic, family caregivers’ responses were mostly similar.

**Conclusion:**

Family caregivers had support needs that were not always met, which was especially the case for spiritual support needs. Healthcare providers should be trained to accommodate support needs and refer to appropriate support services when necessary.

## Key Message

Family caregivers of people with dementia in nursing homes have unmet support needs in their relative’s last week of life, particularly for spiritual support.

## Introduction

Family caregivers of people with dementia often suffer from stress, worry, sadness, and physical burden.^1–3^ Due to the patient’s cognitive deterioration, relationships change, which can lead to complex feelings of loss and grief.^4,5^ In the home care setting, family caregivers of people with dementia report a higher burden of care compared with family caregivers of patients without dementia.^6^ When their relative is admitted to a nursing home, family caregivers can feel relieved, but it can also induce feelings of guilt and shame, and adjustment is needed to a new caregiving role.^1,4,5,7,8^ Therefore, family caregivers should be supported by healthcare providers in nursing homes, which becomes especially important in their relative’s last phase of life.^9–11^ Research on support for family caregivers of people with dementia living in nursing homes is limited, with most studies focusing on the home care context.^12,13^ Furthermore, while existing literature highlights the general experiences of family caregivers and their support needs,^10,14,15^ limited attention is given to the extent to which support needs of family caregivers are actually met. As such, there is limited understanding of how family caregivers’ specific support needs, such as emotional, practical, and spiritual support, are addressed by healthcare providers in the nursing home resident’s last phase of life.

The COVID-19 pandemic exerted a great influence on family caregivers’ experiences and to what extent they felt supported. In the Netherlands, similar to other countries worldwide, the government imposed a visitation ban in nursing homes in March 2020 in order to prevent the virus from spreading, allowing exceptions when the patient was in the terminal phase.^16^ Although the ban was lifted halfway through June 2020, visiting restrictions remained; their nature depending on trends in infections on a national level and on policies and infection rates in individual nursing homes. Family caregivers’ opportunities to connect with their relative were limited, impacting emotional well-being.^17,18^ Contact with healthcare providers, who were under increased pressure, was limited as well, and healthcare providers’ resources to support family caregivers were affected by the visiting restrictions.^19–22^ Therefore, the pandemic was an exceptional time in regard to healthcare providers supporting family caregivers.

This study aimed to (1) gain understanding of family caregivers’ experiences during the last week of life of their relative with dementia in a nursing home and the support they received during this time before or during the COVID-19 pandemic, (2) investigate emotional, practical, and spiritual support needs, differences between the occurrence of these needs, and the extent to which healthcare providers accommodated them, and (3) investigate the relationship between the extent to which the three support types were provided and family caregivers’ overall rating of care for themselves.

## Methods

### Study design

As part of a research project to assess evaluations of care,^23^ a survey was sent to bereaved family caregivers of nursing home residents with dementia. The survey was accompanied by an information letter stating that respondents would participate in a research study by completing and returning the survey.

The Medical Research Ethics Committee of LUMC declared the study exempt from the Medical Research Involving Human Subjects Act (WMO; no. P17.214, 19th October 2017). This article follows the Strengthening the Reporting of Observational Studies in Epidemiology^24^ reporting guidelines.

### Data collection

Participants were recruited through psychogeriatric units of 6 out of 10 long-term care facilities of a healthcare organization in the West of the Netherlands. Initially, data collection took place in January 2019, when one batch of surveys was sent by postal mail to primary contact persons of all residents who had died between March and December 2018. At the start of the pandemic in the Netherlands (March 2020), it was decided to resume data collection to also include experiences during this time. Between August 2020 and May 2022, the survey was sent every three months, in batches, to all primary contact persons of the residents who had died in the preceding months, excluding family caregivers that were bereaved in the preceding six weeks. The data entry and management of returned surveys were facilitated by Castor EDC.^25^

### Measures

The survey was developed in collaboration with the study groups of the Dutch End of Life in Dementia study^26^ and the Empowering Better End-of-Life Dementia Care study.^27^ Formatting some of the questions was guided by the Toolkit of Instruments to Measure End-of-life Care.^28^ Since most items were previously used in other studies, pilot testing the survey was deemed unnecessary. The survey contained questions about the characteristics of the family caregiver and their relative with dementia, the circumstances surrounding the resident’s death and the care they received, and the care the family caregivers received themselves (Supplementary Appendix). The questionnaire was expanded with pandemic-related questions when data collection was resumed in 2020. The questionnaire consisted mostly of closed-ended questions, but for some questions respondents were asked to explain their answer. To gain an understanding of family caregivers’ experiences during their relative’s last week of life, various items were selected relating to the circumstances of the resident’s last week of life (e.g., time spent with relative during the last week, whom were present at the time of death, and whether the number of healthcare professionals was sufficient), as well as items inquiring after the impact of the COVID-19 pandemic on family caregivers’ experiences.

Family caregivers’ needs for emotional, practical, and spiritual support and the extent to which those needs were accommodated were assessed with the question, “In the last week of your relative’s life, how often did the healthcare providers give the kind of 1) emotional support 2) practical support 3) spiritual support you wanted?.” The answering options were “always,” “most of the time,” “sometimes,” “never,” and “I did not want such support/did not need it.” Further, family caregivers’ overall rating of care was assessed with the question “Overall, how would you rate the care that you yourself received in the last week of your relative’s life (and, if applicable, after your relative’s death)?” with the answering options “excellent,” “very good,” “good,” “fair,” “poor,” and “healthcare providers did not provide care to me.”

### Statistical analyses

First, descriptive statistics were used to present the characteristics of the family caregivers and residents, the circumstances of the resident’s last week of life and death, the support that family caregivers received during this time, and the influence of the COVID-19 pandemic on family caregivers’ experiences. Results are presented for the entire sample, as well as separately for the periods before and during the COVID-19 pandemic. The answers to open questions were categorized and described.

Differences between family caregivers’ reported need for emotional, practical, and spiritual support in their relative’s last week of life and between the extent to which these types of support were provided were tested with Pearson’s chi-square tests. Bivariable logistic regression analyses, unadjusted and adjusted for family caregivers’ sex and age, were performed to assess associations between the emotional, spiritual, and practical support that was provided (independent variables) and family caregivers’ overall rating of care for themselves (dependent variable).

Responses to the dependent variable (overall rating of care) were dichotomized to account for a ceiling effect to a high rating (“excellent” or “very good”) and a medium or low rating (“good,” “fair,” or “poor”). Respondents also had the option to answer with “healthcare providers did not provide care to me” for when they considered the question to be inapplicable. There was a possibility that respondents used this answering option to indicate they did not receive care even though they had wanted this rather than using the options for fair or poor care. To account for this, when family caregivers indicated a need for at least one type of support but a low frequency thereof, their response was classified as “medium or low rating” rather than omitting their response. In other cases, responses were excluded from the denominator. This was the case when family caregivers indicated not needing two or more support types or when responses were inconsistent (e.g., reporting that support was always provided, but also that healthcare providers did not provide care).

For the independent variables (frequency with which emotional, practical, and spiritual support were provided), we omitted responses of the answering option “I did not want such support/did not need it” from the denominator, as the question was not applicable to those family caregivers. As this decreased the sample, the responses were dichotomized to “high frequency” (“always” and “most of the time”) and “low frequency” (“sometimes” and “never”).

A two-tailed *p* value of <0.05 was considered significant. All analyses were performed in SPSS (IBM, version 29, 2022).

## Results

The overall response rate was 55% (165/301 family caregivers). Before the pandemic, the questionnaire was completed by 50 of 87 (57%) bereaved family caregivers, and during the pandemic by 115 of 214 (54%). Most family caregivers were a child of the patient (73%). The mean age of respondents was 62 years (range: 41–88). The mean age of the residents at the time of death was 87 years (range: 66–100; Table 1).

**Table 1.** Characteristics of Family Caregivers and Residents as Reported by Family Caregivers

	Total (*N* = 165)		Pre-COVID (*N* = 50)		COVID (*N* = 115)	
		*n*		*n*		*n*
Family caregiver						
Sex, %						
Female	73	119	78	39	71	80
Male	27	44	22	11	29	33
Age in years, mean [SD]	62 [10]	163	60 [10]	50	63 [10]	113
Relationship to the resident, %						
Child^a^	73	120	74	37	72	83
Spouse	16	26	12	6	17	20
Cousin/niece/nephew	5	9	6	3	5	6
Sibling^b^	4	6	4	2	3	4
Granddaughter	1	1	2	1	-	0
Nonfamilial relationship (friend/neighbor)	2	3	2	1	2	2
Resident						
Sex, %						
Female	66	108	72	36	63	72
Male	35	57	28	14	37	43
Age at the time of death in years, mean [SD]	87 [7]	162	85 [7]	49	88 [7]	113
Place of birth,^c^ %						
The Netherlands	95	109	—	—	95	109
Other	5	6	—	—	5	6
Cause of death (multiple answers possible),^c^ %						
Advanced dementia	49	56	—	—	49	56
Stopped eating and drinking	40	46	—	—	40	46
Heart failure	12	14	—	—	12	14
Complications after a fall	10	12	—	—	10	12
COVID-19 (symptoms)	10	12	—	—	10	12
Difficulty swallowing	6	7	—	—	6	7
Pneumonia	6	7	—	—	6	7
Infection(s)	4	5	—	—	4	5
Stroke	4	4	—	—	4	4
Cancer	2	2	—	—	2	2
Doctor did not disclose cause of death	6	7	—	—	6	7
I do not know	4	4	—	—	4	4
Doctor did not know cause of death	4	5	—	—	4	5
Other^d^	2	2	—	—	2	2

SD, standard deviation.

^a^ Includes children-in-law (*n* = 6).

^b^ Includes siblings-in-law (*n* = 2).

^c^ The item was included in the questionnaire during COVID only.

^d^ Arterial occlusion of the leg (*n* = 1), neurological (seizures; *n* = 1).

### The resident’s last week of life and their death

During the pandemic, the resident died alone more often (32%) than before (20%; Table 2). Sixty-one percent of all respondents had not expected that their relative was going to die a month before, and almost a quarter (23%) reported that no one alerted them that death was near. This did not differ substantially between respondents before and during the pandemic. Most family caregivers believed that the number of available healthcare providers was certainly sufficient both before (59%) and during the pandemic (63%). Of all respondents, the majority (87%) were satisfied in every respect or with the main elements of the communication with healthcare providers, with more family caregivers being satisfied in every respect during the pandemic (63%) than before (42%). Further, during the pandemic, more family caregivers reported full agreement between all people involved on the resident’s treatment and care in their relative’s last week of life (76%) than before the pandemic (55%).

**Table 2.** Resident’s Last Week of Life and Death as Reported by Family Caregivers

	Total (*N* = 165)		Pre-COVID (*N* = 50)		COVID (*N* = 115)	
		*n*		*n*		*n*
Whom were present when your relative died? %						
My relative died alone	28	46	20	10	32	36
Family caregivers were present	41	67	48	24	38	43
Healthcare providers were present	19	31	20	10	18	21
Both family caregivers and healthcare providers were present	12	20	12	6	12	14
Were you able to see/visit your relative in the week before they died?^a^ %						
Yes	92	106	—	—	92	106
No	8	9	—	—	8	9
During the last week of his or her life, approximately how many hours did you spend with your relative? mean [SD]	28 [32]	161	29 [27]	47	28 [33]	114
If you think back to one month before your relative died, do you feel like at that time you expected that he/she was going to die? %						
Yes	34	55	38	19	32	36
No	61	100	58	29	62	71
I do not know	6	9	4	2	6	7
Did anyone alert you or other family caregivers shortly before death when your relative was about to die? %						
Yes	77	125	72	36	79	89
No	23	38	28	14	21	24
Was the place where your relative died their familiar “home” or had it become their familiar home? %						
Yes	52	85	52	26	52	59
Partly	29	48	36	18	26	30
No	19	31	12	6	22	25
Do you feel that, in the last week of your relative’s life, the number of professional healthcare providers available was sufficient? %						
Yes, plenty (certainly sufficient)	62	98	59	29	63	69
Yes, (only) just sufficient	30	48	35	17	28	31
No, there was a shortage of professional healthcare providers	8	12	6	3	8	9
Has your relative been to hospital during the last week of their life? %						
Yes (emergency department)	1	2	2	1	1	1
No	99	162	98	49	99	113
In the last week of life, was there any medical procedure or treatment that happened to your relative that was inconsistent with his/her previously stated wishes? %						
Yes	4	6	4	2	4	4
No	87	143	80	40	90	103
I do not know	10	16	16	8	7	8
Are you satisfied with how the communication with healthcare providers went (discussions on future care, goals of treatment, and care in the last phase of life)? %						
Satisfied in every respect	57	91	42	20	63	71
Satisfied about the main elements	30	48	42	20	25	28
Neutral	5	8	4	2	5	6
Not satisfied	7	11	10	5	5	6
Did not talk to healthcare providers, while I would have wanted to	1	1	—	0	1	1
Did not talk to healthcare providers and I do not think that it was needed	1	2	2	1	1	1
To what degree did all persons involved in the treatment(s) and care [nursing home staff and (other) family members], agree about the best treatment(s) in the last week of life? %						
Fully agreed	70	112	55	27	76	85
Agreed on major issues	26	42	41	20	20	22
Did not agree	4	7	4	2	5	5

SD, standard deviation.

^a^ The item was included in the questionnaire during COVID only.

### Family caregivers’ experiences of support for themselves in their relative’s last week of life

Nineteen percent of the family caregivers reported that they had an unpleasant experience in their relative’s last week of life, with no large difference between before and during the pandemic (Table 3). The reported unpleasant experiences concerned the care for their relative, the communication with and between healthcare providers, the support the family caregivers received (or a lack thereof), or the pandemic (visiting restrictions and receiving little time to empty their relative’s room after their death). During the pandemic, more family caregivers indicated having been offered grief and bereavement counseling from nursing home staff after their relative’s death (43%) than before the pandemic (33%). How the family caregivers rated the overall support they received did not differ between before and during the pandemic, with 79% rating this as “excellent,” “very good,” or “good.” When support was rated as “poor” or “fair,” the explanations regarded a lack of support and attention before and after their relative’s death, ineffective communication, having missed a fixed point of contact, and the visiting restrictions. Similar topics were reported in the answers to the question “How could healthcare providers have looked after you better?.” Other topics addressed were staffing issues, practical support, provision of spiritual support, and taking family caregivers’ perspectives into serious consideration.

**Table 3.** Family Caregivers’ Experiences of Support for Themselves in Their Relative’s Last Week of Life (*N* = 165)

	Total		Pre-COVID		COVID		Total		Pre-COVID		COVID		Total		Pre-COVID				COVID	
	%	*n*	%	*n*	%	*n*	%	*n*	%	*n*	%	*n*	%	*n*	%		*n*		%	*n*
Were you in need of … …	… emotional support? (*N* = 162)						… practical support? (*N* = 162)						… spiritual support? (*N* = 161)							
Yes	75	121	77	37	74	84	64	104	69	33	62	71	37	59	30		14		40	45
No	25	41	23	11	26	30	36	58	31	15	38	43	63	102	70		33		61	69
If in need: How often, in the last week of life of your relative, did the healthcare providers offer the kind of …	… emotional support you wanted? (*N* = 121)						… practical support you wanted? (*N* = 104)						… spiritual support you wanted? (*N* = 59)							
Always	31	37	27	10	32	27	36	37	30	10	38	27	14	8		14	2	13		6
Most of the time	32	39	35	13	31	26	15	16	18	6	14	10	9	5		7	1	9		4
Sometimes	26	32	24	9	27	23	25	26	24	8	25	18	19	11		21	3	18		8
Never	11	13	14	5	10	8	24	25	27	9	23	16	59	35		57	8	60		27
Have you had any unpleasant experience with care for your relative or for you?																				
Yes	19	31	17	8	20	23														
No	81	130	83	40	80	90														
Overall, how would you rate the care that you yourself received in the last week of your relative’s life (and, if applicable, after your relative’s death)?																				
Excellent	25	40	23	11	26	29														
Very good	27	43	28	13	27	30														
Good	28	44	30	14	27	30														
Fair	5	8	6	3	4	5														
Poor	3	5	4	2	3	3														
Healthcare providers did not provide care to me	13	20	9	4	14	16														
Since your relative’s death, did anyone from the nursing home contact you to offer grief and bereavement counseling or support services?																				
Yes	40	65	33	16	43	49														
No	60	96	67	32	57	64														

Both before and during the pandemic, family caregivers more commonly reported needing emotional (75%) and practical (64%) support in their relative’s last week of life compared with spiritual support (37%). The difference in support needs between support types was statistically significant (*p* < 0.001). Seventeen percent reported not needing any of the support types, and 29% reported a need for all three. When respondents had support needs, a significant difference was found between the support types in the frequency with which the support was provided (*p* < 0.001). Respondents reported more commonly that emotional and practical support were “always” or “most of the time” provided (63% and 51%, respectively) compared with spiritual support (22%).

For the logistic regression analyses, the 20 responses to the question on overall rating of care indicating “healthcare providers did not provide care to me” were examined in relation to the responses of the same respondents to the questions on emotional, practical, and spiritual care. This resulted in 11 responses being excluded from the denominator and the remaining 9 being reclassified to “medium or low rating.” The analyses showed that family caregivers were more likely to rate the overall care they received as “excellent” or “very good” when they reported a higher frequency of emotional (odds ratio [OR] = 20, *p* < 0.001), practical (OR = 7, *p* < 0.001), or spiritual support provided (OR = 29, *p* = 0.002; Table 4).

**Table 4.** Associations of Experienced Frequency of Emotional, Spiritual, and Practical Care by Family Caregivers with Their Overall Rating of Care

Logistic regression analyses Dependent variable: high rating of care vs. low or medium rating of care						
	Unadjusted			Adjusted^a^		
	OR	95% CI	*p* value	OR	95% CI	*p* value
Emotional support^b^	20	[8–54]	<0.001	19	[7–51]	<0.001
Spiritual support^c^	29	[3–243]	0.002	29	[3–256]	0.002
Practical support^d^	7	[3–16]	<0.001	7	[3–18]	<0.001

CI, confidence interval; OR, odds ratio.

^a^ Adjusted for sex and age of family caregiver.

^b^ *N* = 117.

^c^ *N* = 57.

^d^ *N* = 101.

### Impact of the COVID-19 pandemic on family caregivers’ experiences

Almost half of the respondents reported that the pandemic influenced how they felt (42%), and over a third (35%) reported it influenced their experience of grief (Table 5). Explanations showed that, for some, visiting restrictions induced feelings of guilt and a belief the restrictions had hastened death. Some expressed relief their relative did not suffer from the pandemic’s consequences any longer. Over a third of the respondents (38%) reported that the pandemic impacted the social support they received. Explanations regarded them receiving fewer visits from family and friends, missing physical contact, and the societal lockdown resulting in the closure of leisure clubs and the inability to attend events and make outings. The impact of the pandemic on farewell rituals was reported most commonly, with 63% of the respondents reporting this to be the case. Explanations related to the restricted number of people that could attend the funeral and the lack of a get-together with attendees afterwards. The majority regarded this negatively, but some family caregivers considered it to be valuable as it increased intimacy.

**Table 5.** Impact of the COVID-19 Pandemic on Family Caregivers’ Experiences at Their Relative’s End of Life (*N* = 115)

	%	*n*
Do you think that COVID-19 has impacted on how you are feeling?		
Yes	42	47
No	58	64
In particular, do you think COVID-19 has impacted on your experience of grief?		
Yes	35	39
No	65	73
Has COVID-19 affected your social support?		
Yes	38	41
No	62	68
Do you think that COVID-19 has impacted the end-of-life care your relative received?		
Yes	40	45
No	60	67
Do you think that COVID-19 affected your farewell rituals?		
Yes	63	70
No	37	41

## Discussion

Family caregivers had support needs that were addressed to some extent but not fully. Emotional and practical support needs were more often reported than spiritual support needs. When in need of support, emotional and practical support were provided more frequently compared with spiritual support. All three types of support contributed to family caregivers’ overall rating of the care they received. Responses of family caregivers before and during the pandemic were mostly similar.

For some family caregivers in the current study, practical, emotional, and in particular spiritual support needs remained unmet. This corresponds with literature from other countries reporting spiritual support to be limited in practice,^29–31^ despite its importance having been highlighted in guidelines and in empirical studies.^5,9,32–34^ Nurses in nursing homes play a pivotal role in accommodating family caregivers’ needs, as they generally know family caregivers well and are in a position to recognize their needs, but they may not feel confident to provide spiritual support with little formal training and education in this area.^30,35,36^ However, in an ethnographic study in Dutch nursing homes, many examples were observed of nurses providing spiritual support to residents in an *ad hoc* informal manner, which may be unrecognized as spiritual support by themselves.^37^ Spiritual counselors can empower nurses in their skills by offering training and education and by being available for referrals when family caregivers’ needs exceed the nurses’ capabilities. Training should aim to enhance nurses’ skills in identifying and exploring family caregivers’ signals that may indicate spiritual support needs.^38^ Further research may contribute to an understanding of the specific spiritual support needs of family caregivers and how those needs are best accommodated.

In this study, more than half of the family caregivers did not expect their relative was going to die one month before the death. This is in line with literature highlighting that the end of life of people with dementia can be hard to predict.^39–41^ More notable is the finding of almost a quarter of family caregivers reporting not having been alerted that death was near shortly before the death. This is surprising, as a Dutch survey study among physicians working in long-term care facilities demonstrated that only 11% of deaths among people with dementia were considered unexpected.^42^ Also, elderly care physicians who work in Dutch nursing homes are extensively trained to provide end-of-life care and to communicate with family caregivers.^43,44^ The findings of the current study may in part be explained by the difficulty of emotionally preparing for a relative’s death.^45^ A cross-sectional mixed-methods study found that only 29% of family caregivers of people with dementia in residential and nonresidential settings felt emotionally prepared for their relative’s death.^46^ As such, it could be that the impending death of a relative may be hard to process, regardless of whether healthcare providers have addressed it. This may especially be the case when the resident is not suffering from burdensome symptoms and is, for instance, still able to communicate.^46,47^ To better understand these processes, interview and observation studies should be conducted to explore what conversations take place between healthcare providers and family caregivers during the resident’s last week of life and how these are processed by family caregivers.

The finding that family caregivers’ responses were mostly similar between before and during the pandemic might be unexpected given the literature on the enormous impact of the pandemic on family caregivers’ experiences.^48–52^ This might partly be explained by this study’s focus on the resident’s last week of life, as restrictions were commonly loosened when residents entered the dying phase. Notably, family caregivers were more satisfied with the communication with healthcare providers during the pandemic and more often did all people involved agree about the best treatment. The threat that a COVID-19-infection imposed to older adults, the shortage of hospital beds, and the attention Dutch media paid to this may have prompted an increased sense of urgency regarding advance care planning, both for healthcare providers and family caregivers,^53,54^ facilitating more and quicker alignment between them.^55,56^ This is in line with the findings of a Dutch qualitative survey among physicians working in nursing homes, which indicated that the pandemic triggered additional advance care planning conversations.^54^

### Strengths and limitations

A strength of this study is that it adds to an understanding of how family caregivers of people with dementia in their last week of life experience the care they receive themselves. The distinction that was made between emotional, practical, and spiritual support contributes to this understanding. Further, including family caregivers before and during the pandemic showed that family caregivers’ reported experiences are largely independent of the context of the pandemic.

This study also has limitations. Only limited significance can be attached to the associations between the frequency of support family caregivers received and their overall rating of care due to the wide confidence intervals. Further, this study provides little insight into support for family caregivers after their relative’s death. The one item that addresses such support inquiries after grief and bereavement counseling specifically. It is possible that family caregivers were supported by healthcare providers after their relative’s death, either on their own initiative by visiting the nursing home or by being invited for a follow-up conversation. However, they might not have considered this as part of grief and bereavement counseling. Interview and observation studies may help to understand what kind of contact is established with family caregivers after their relative’s death and to what extent family caregivers feel supported by this.

### Conclusion

Emotional, practical, and especially spiritual support were not always provided to family caregivers in the last week of the life of their relative, all of which were of importance to their overall rating of the care they received themselves. Healthcare providers should be trained to identify and accommodate those needs and to be able to refer family caregivers to appropriate support services when necessary. Future qualitative and observational studies are needed for a better understanding of family caregivers’ specific support needs, how well these are currently met, and opportunities for improvement.
