# Supplementary material for: Supporting Family Caregivers of Nursing Home Residents with Dementia in Their Last Week of Life: A Survey Among Bereaved Family Caregivers
Source: Palliat Med Rep. 2025 Mar 5;6(1):105–15. doi: 10.1089/pmr.2024.0088 (PMC11947660; doi:10.1089/pmr.2024.0088)
Supplement: Supplementary Appendix [file pmr.2024.0088_supplementaryappendix.docx]

**Appendix A**

Survey questions addressed in the study *“Support for family caregivers during the last week of life of their relative with dementia in a nursing home: survey among bereaved family caregivers”*.

The survey was developed in collaboration with the study groups of the Dutch End of Life in Dementia (DEOLD) study^1^ and the Empowering Better End-of-Life Dementia Care (EMBED-care) study.^2^ Formatting of some of the questions was guided by the Toolkit of Instruments to Measure End-of-life Care.^3^ Self-developed questions were translated by a professional translator.

**General questions about your family member / loved one**

**1.** Was your family member / loved one:

- male
- female

**2.** What was the age at the time of death?

…………………years

**3.** Your family member / loved one was born in:

 the Netherlands

 other, specify:……………………..

**4.** What was exactly your relationship to your family member / loved one? I was his or her:

- spouse
- partner otherwise
- son or daughter
- sibling
- other family connection, I was his or her:……………………..
- representative appointed by a judge (curator, mentor, administrator)
- other, specify:……………………….

**At the time of death**

**5.** If you think back to one month before your family member / loved one died, do you feel like at that time you expected that he/she was going to die?^1^

- yes
- no
- don’t know

**6.** Did anyone alert you or your (other) family shortly before death when your family member / loved one was about to die?^1^

- yes
- no
- don’t know

**7.** Was the place where your family member / loved one died their familiar “home” or had it become their familiar “home”?

- yes
- partly
- no

**8.** Whom were present when your family member / loved one died? *(multiple answers possible)*^1^

- you yourself
- other family, or other loved one(s)
- nursing home physician(s)
- nurse(s) or nurse aid(s)
- pastor, chaplain, referent
- someone else, specify: ……………………….
- nobody

**9.** Has your family member / loved one been to hospital during the **last week** of their life?

- yes, he or she was hospitalized
- yes, he or she was admitted to an intensive care unit
- yes, to the hospital emergency department (ED)
- yes, to the outpatient clinic
- no

**10.** During the last week of his or her life approximately how many hours did you spend with your family member / loved one? *(if you are not sure, try to provide an estimate)*^1^

approximately ………………… hours

**11.** What was the cause of death?

- COVID-19/Coronavirus symptoms
- difficulty swallowing
- pneumonia
- heart failure
- infection(s)
- cancer
- complications after a fall
- stopped eating/drinking
- stroke
- advanced dementia
- other, specify:……………….
- I don’t know
- doctor did not disclose cause of death
- doctor did not know cause of death

**12.** Were you able to see/visit your family member / loved one in the week before they died?^2^

Please describe

- yes
- no

If yes or no, please describe:…………………………………………………………………..

………………………………………………………………………………………………..

**Satisfaction with care during the last week of life**

**13.** Do you feel that, in the last week of life, the number of healthcare providers available was sufficient?^1^

- yes, plenty (certainly sufficient)
- yes, (only) just sufficient
- no, there was a shortage, specifically, a shortage of:……………………………………………….

**14.** In that last week, was there any medical procedure or treatment that happened to your family member / loved one that was inconsistent with his/her previously stated wishes?^1^

- yes
- no
- I don't know: previously stated wishes were either not known or too little known

**15.** To what degree did all persons involved in the treatment(s) and care (healthcare providers and (other) family members) **agree** about the best treatment(s) and care in the last week of life?^1^

- fully agreed
- agreed on major issues
- did not agree, opinions did **not** agree between:

…………………… and ………………….

**16.** Are you satisfied with how the communication with healthcare providers went (discussions on future care, goals of treatment, and care in the last phase of life, in the last week)?^1^

- satisfied in every respect
- satisified about the main elements
- neutral
- not satisfied, because: ………………………………..
- did not talk to healthcare providers while I would have wanted to
- did not talk to healthcare providers and I did not think it was needed

**Questions about care provided to you, and your experiences in general**

**17.** Since your family member’s / loved one’s death, did anyone from the nursing home contact you to offer grief and bereavement counselling or support services?^1^

- yes
- no

**18.** In the last week of your family member's / loved one's life, how often did healthcare providers give the kind of **emotional support** you wanted?^3^

- always
- most of the time
- sometimes
- never
- I did not want / need emotional support

**19.** In the last week of your family member's / loved one's life, how often did healthcare providers give the kind of **spiritual support** (philosophical or religious care) you wanted?^3^

- always
- most of the time
- sometimes
- never
- I did not want / need mental support

**20.** In the last week of your family member's / loved one's life, how often did healthcare providers give the kind of **practical support** you wanted (e.g., organizing things)? ^3^

- always
- most of the time
- sometimes
- never
- I did not want / need practical support

**21.** Overall, how would you rate the care that **you yourself** received from the healthcare providers during the last week of your family member's / loved one's life (and, if applicable, after the death of your family member / loved one)?^1^

- excellent
- very good
- good
- fair, because…………………………….
- poor, because:…………………………….
- the healthcare providers did not take care of me during the last week

**22.** Have you had any **unpleasant experience** with care for your family member / loved one or for you? *(please describe the experience and specify who could have done better)*^1^

- no, I did not have any unpleasant experiences
- yes, specify: ………………………………………………..………………………………………………..………………………………………………..………………………………………………..………………………………………………..………………………………………………..………………………………………………..………………………………………………..………………………………

Who could have done better? …………………………………………………………………….

**23.** In what way could healthcare providers have taken better care of you? *(e.g. what kind of care did you lack but wish you had been given or do you have any suggestions for the healthcare providers?)*

………………………………………………..………………………………………………..………………………………………………..………………………………………………..……………………

**Questions regarding COVID-19**

**24.** Do you think that COVID-19 has impacted on how you are feeling?^2^

- yes
- no

Please describe: …………………………………………………………………………………………………………………………………………………………………………………………………………………………

**25.** In particular, do you think COVID-19 has impacted on your experience of grief? ^2^

- yes
- no

Please describe: …………………………………………………………………………………………………………………………………………………………………………………………………………………………

**26.** Has COVID-19 affected your social support? ^2^

- yes
- no

Please describe: …………………………………………………………………………………………………………………………………………………………………………………………………………………………

**27.** Do you think that COVID-19 has impacted the end-of-life care your family member/ loved one received? *(e.g. the different kinds of care or different healthcare providers, visiting bans or restrictions)*

- yes
- no

Please describe: …………………………………………………………………………………………………………………………………………………………………………………………………………………………

**28.** Do you think that COVID-19 affected your farewell rituals? *(e.g. restrictions at funeral/gatherings)*

- yes
- no

Please describe: …………………………………………………………………………………………………………………………………………………………………………………………………………………………

**Conclusion**

**29.** What is your gender?

- male
- female

**30.** Wat is your age?

………………….. years

**31.** Do you have any additional thoughts about the survey?

- yes

………………………………………………..………………………………………………..………………………………………………..………………………………………………..………………………………………………..………………………………………………..………………………………………………..………………………………………………..………………………………

- no

**References**

1. van der Steen JT, Ribbe MW, Deliens L, et al. Retrospective and prospective data collection compared in the Dutch End Of Life in Dementia (DEOLD) study. Alzheimer Dis Assoc Disord 2014;28(1):88-94; doi:10.1097/WAD.0b013e318293b380.

2. Sampson EL, Anderson JE, Candy B, et al. Empowering Better End-of-Life Dementia Care (EMBED-Care): A mixed methods protocol to achieve integrated person-centred care across settings. Int J Geriatr Psychiatry 2020;35(8):820-832; doi:10.1002/gps.5251.

3. Toolkit of instruments to measure end of life care: after-death bereaved family interview: nursing home version. Brown University: Providence, RI; 2000. Available from: https://www.chcr.brown.edu/pcoc/linkstoinstrumhtm.htm.
